# High-fructose and high-fat diet-induced insulin resistance enhances atherosclerosis in Watanabe heritable hyperlipidemic rabbits

**DOI:** 10.1186/s12986-015-0024-3

**Published:** 2015-08-12

**Authors:** Bo Ning, Xiaoyan Wang, Ying Yu, Ahmed Bilal Waqar, Qi Yu, Tomonari Koike, Masashi Shiomi, Enqi Liu, Yifei Wang, Jianglin Fan

**Affiliations:** Department of Molecular Pathology, Interdisciplinary Graduate School of Medicine and Engineering, University of Yamanashi, Yamanashi, Japan; Biomedicine Research and Development Center, Jinan University, Guangzhou, China; Institute for Experimental Animals, Kobe University School of Medicine, Kobe, Japan; Department of Pathology, Xi’an Medical University, Xi’an, China; Research Institute of Atherosclerotic Disease and Laboratory Animal Center, Xi’an Jiaotong University School of Medicine, Xi’an, China

## Abstract

**Background:**

Individuals with insulin resistance and resulting impaired glucose tolerance along with type 2 diabetes showed an increased prevalence of atherosclerosis. Our aim in this study was to address whether diet-induced insulin resistance plays any roles in the development of aortic and coronary atherosclerosis in hyperlipidemic rabbits.

**Methods:**

We fed Watanabe heritable hyperlipidemic (WHHL) rabbits with a high-fructose and high-fat diet (HFFD) with restricted normal calories and compared the lesions of both aortic and coronary atherosclerosis with those of control WHHL rabbits fed a normal chow diet.

**Results:**

HFFD-fed WHHL rabbits showed insulin resistance and impaired glucose tolerance accompanied by elevated plasma lipid levels and accumulation of adipose tissue even though their body weight was unchanged compared to the control rabbits. At 8 weeks, the aortic gross lesion area of HFFD-fed WHHL rabbits was increased by 40 % over the controls and their lesions were characterized by increased number of macrophages and smooth muscle cells. At 16 weeks, the lesions of HFFD-fed WHHL rabbits showed more advanced lesions such as lipid core formation and calcification. In addition, coronary atherosclerosis was significantly increased in HFFD-fed WHHL rabbits.

**Conclusions:**

These results suggest that insulin resistance accelerates lesion formation of atherosclerosis.

**Electronic supplementary material:**

The online version of this article (doi:10.1186/s12986-015-0024-3) contains supplementary material, which is available to authorized users.

## Introduction

Insulin resistance (IR) is frequently associated with many metabolic diseases such as obesity, hypertriglyceridemia, type 2 diabetes, and metabolic syndrome [1]. Individuals with underlying IR along with impaired glucose tolerance also have an increased prevalence of atherosclerosis and coronary heart disease [2, 3]. However, whether IR is directly involved in the development of atherosclerosis has not yet been fully understood [4, 5]. IR is simply defined as impaired response to insulin of the liver, adipose tissue and skeleton muscles due to impairments of the insulin signaling pathway [3]. In experimental animals, IR can be induced by a diet containing high-fructose and/or high-fat. Such diet feeding can result in IR in rats [6, 7] but not in low-density lipoprotein (LDL) receptor-deficient mice [8]. Although rats are useful for the study of IR, hyperinsulinemia, and hyperglycemia, they are resistant to diet-induced hypercholesterolemia and atherosclerosis because their lipoprotein metabolism is largely different from humans. It is well known that unlike humans, rats as well as mice, are deficient in cholesteryl ester transfer protein (CETP), an important regulator of lipoprotein metabolism and their major lipoproteins are high-density lipoproteins (HDLs) rather than LDLs. Therefore, it is difficult to use these animal models to elucidate the relationship between IR and atherosclerosis.

To examine whether IR plays any roles on atherosclerosis, we performed the current study using Watanabe heritable hyperlipidemic (WHHL) rabbits. Like humans but unlike rodents (rats and mice), rabbits have abundant plasma CETP and their lipoprotein profiles are LDL-rich [9]. WHHL rabbits are genetically deficient in low-density-lipoprotein (LDL) receptor due to a spontaneous 4-amino-acid deletion in the cysteine-rich ligand-binding domain in exon 4 of the LDL receptor and develop hyperlipidemia and spontaneous atherosclerosis on a normal chow diet [10, 11]. WHHL rabbits have been extensively used for the study of hypercholesterolemia and atherosclerosis [12, 13]. In the current study, we fed WHHL rabbits with a diet containing high fructose and fat (HFFD) in attempt to elucidate the roles of IR on early-stage atherosclerosis and advanced atherosclerosis. Our results showed that even when consuming normal numbers of calories similar to the normal chow diet, an HFFD induces IR and enhances the development of aortic and coronary atherosclerosis in WHHL rabbits.

## Methods

### Animals and diets

Watanabe heritable hyperlipidemic (WHHL) MI rabbits [14] (male, 3 months old) provided by Kobe University, Japan, were randomly divided into two groups. The control group (n = 15) was fed a standard chow diet (CR-3, CLEA Japan), which contains 17.3 % protein, 3.9 % vegetable fat, 13.6 % fiber, 8.7 % ash, 48.9 % nitrogen-free extract, and 7.6 % moisture by weight, and its physiological fuel value is approximately 2.997 kcal/g. Control WHHL rabbits (2.0 ~ 2.5 kg BW) consumed about 70 ~ 90 g chow diet daily, equivalent to average 210 ~ 270 kcal/day/animal. The high-fructose and high-fat diet (HFFD) group (n = 14) was fed a chow diet supplemented with 30 % fructose and 10 % coconut oil (91 % saturated fatty acids by weight) because HFFD diet can induce more prominent insulin resistance than a diet containing fructose alone. The HFFD was prepared by Oriental Yeast Com. Tokyo. Compared to a standard chow diet, HFFD was rich in sugar and fat but with relatively less protein and fibers. To avoid the excess gain of body weight induced by HFFD, which may complicate the experiments, we fed HFFD group with restricted diets containing approximately similar calories as the control group for 8 weeks (designated as a “short term” to evaluate the early-stage atherosclerosis) and 16 weeks (designated as a “long term” to evaluate advanced lesions of atherosclerosis). The body weight and daily food consumption were monitored during the experiment. All rabbits were housed in individual cages in a room with controlled temperature (22-24 °C) on a 12 h light/dark cycle. This study was approved by the Animal Use and Care Committee of the University of Yamanashi and also conformed to the Guide for the Care and Use of Laboratory Animals published by the US National Institutes of Health.

### Analysis of plasma lipid and glucose metabolism

During the experiment, plasma total cholesterol (TC), triglycerides (TG) and high-density-lipoprotein cholesterol (HDL-C) were determined weekly using Wako assay kits (Wako Pure Chemical Industries, Osaka, Japan) [15]. For the analysis of lipoprotein profiles, plasma lipoproteins were isolated by ultracentrifugation and resolved by electrophoresis in 1 % agarose universal gels (Helena Laboratories, Saitama, Japan) [16]. Plasma lipoproteins were also analyzed using high-performance liquid chromatography (HPLC) by Skylight Biotech, Inc. (Tokyo, Japan) [17]. We measured the rate of VLDL secretion in fasting animals *in vivo* using Triton WR-1339 [18]. Plasma levels of lipid peroxidation products were measured using a TBARS assay kit (Cayman Chemical Co., Ann Arbor, MI).

To examine the deleterious effects of HFFD on glucose metabolism and insulin response, we performed intravenous glucose tolerance test (IVGTT) and intravenous insulin tolerance test (IVITT) at 4, 8 and 16 weeks using a method reported previously [19]. The rabbits were injected with glucose solution (0.6 g/kg body weight) intravenously after 16 h of fasting, and then blood samples were drawn at 5, 10, 15, 20, 30, 45, 60, 75 and 120 min. For IITT, after the intravenous injection of insulin (1 U/kg body weight, Novo Nordisk Pharmaceutical Co., Tokyo, Japan), blood samples were collected at 15, 30, 45, 60, 90 and 120 min. The glucose and insulin concentrations were measured using Wako glucose assay kits and rabbit insulin ELISA kits (Shibayagi Co., Ltd., Gunma, Japan).

### Pathological analysis

Rabbits were euthanized by an overdose injection of sodium pentobarbital (100 mg/kg) and the adipose tissue (both subcutaneous and visceral adipose) was collected and weighed wet. For histological analysis, adipose tissue, along with liver and pancreas, was fixed in 10 % buffered formalin. After paraffin-embedding, sections (3 μm thick) were routinely cut and stained with hematoxylin and eosin (H&E). Adipocyte size was evaluated under a light microscope using H&E-stained sections, as described previously [19]. Briefly, more than 300 adipocytes from each section were randomly measured for cellular diameter by two observers blindly, using an image analysis system (Winroof ver 6.4, Mitani Co., Tokyo) [19]. The average diameter of adipocytes in each group was calculated and the cell size distribution was analyzed and expressed as a percentage. Islet number and islet area on the sections immunohistochemically stained with insulin monoclonal antibody were calculated as described previously [20].

### Analysis of aortic and coronary atherosclerosis

The aorta and heart were isolated from each rabbit. Aortic trees were opened and fixed in 10 % buffered formalin. After formalin fixation, the whole aortas were stained with Sudan IV and photographed for evaluation of the gross size of atherosclerotic lesions [14]. The surface sudanophilic area of each aorta segment was measured using an image analysis system. For histological analysis, serial sections (3 μm thick) were stained with H&E, Elastica van Gieson (EVG) and immunohistochemically stained with RAM11 (working dilution 400X) and HHF35 (200X) monoclonal antibodies against macrophages and smooth muscle α-actin (Dako) [14]. The microscopic lesion area, macrophages and SMCs in the lesions were quantified and early-stage lesions and advanced lesions were classified and analyzed using an image analysis system [14]. Advanced lesions refer to those which to contain a typical fibrotic cap and a lipid or necrotic core with or without calcification [14]. Rabbit hearts were sectioned and coronary atherosclerosis was analyzed and expressed as stenosis %, as described previously [15].

### Real-time reverse transcriptase-polymerase chain reaction (RT-PCR)

Total RNA from the liver was isolated using Trizol reagent (Invitrogen, Life Technologies, Inc., Carlsbad, CA) and then mRNA expression of nuclear factor-like 2 (Nrf2), superoxide dismutase 1 (SOD1) and microsomal triglyceride transfer protein (MTTP) was analyzed by real-time reverse transcriptase (RT)-polymerase chain reaction (PCR) [14]. The panel of primers used for analyzing the gene expression is shown in Additional file 1: Table S1.

### Statistical analysis

Statistical analyses were performed using SPSS 12.0 software. All data are expressed as the mean ± SD. Statistical significance data was determined for parametric data by Student’s *t*-test and for non-parametric data by Mann-Whitney *U*-test. In all cases, a *p* value of less than 0.05 was considered statistically significant.

## Results

### Effect of HFFD on plasma lipids and lipoproteins

There was no change in body weight between the two groups (2.28 ± 0.32 in the control *vs* 2.32 ± 0.21 kg in HFFD at 0w; 2.40 ± 0.28 *vs* 2.46 ± 0.24 kg at 8w, and 2.56 ± 0.15 *vs* 2.62 ± 0.14 kg at 16 w) as they consumed similar numbers of calories of each diet (Additional file 1: Figure S1). It should be noted that this HFFD contained relative shortage of other components (such as proteins and fibers) compared to the standard chow diet. In the current study; we fed both groups with restricted amount of each diet containing equal numbers of calories to minimize the HFFD effect on the body weight.

**Fig. 1:**
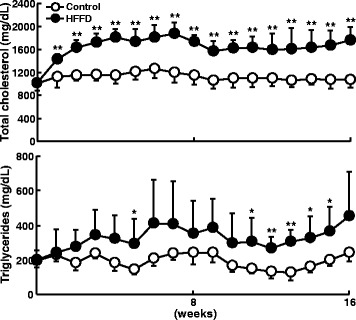
Plasma total cholesterol (top) and triglycerides (bottom). Data are expressed as the mean ± SD. N = 9-15 for each group. **P < 0.01 or *P < 0.05 vs. the control group

**Fig. 2:**
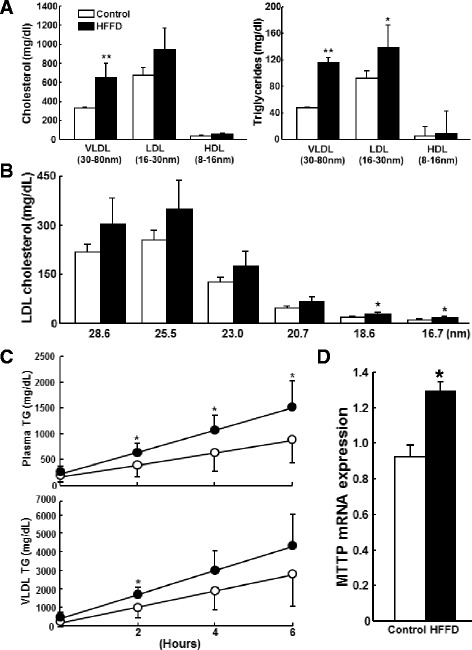
**a-b** Quantitative analysis of VLDL, LDL and HDL contents (**a**) and different-sized LDL fractions (**b**). Plasma lipoproteins (at 8 weeks) were fractionated according to their size by HPLC and lipid contents of each fraction were quantified as described in the Methods. Data are expressed as the mean ± SD. n = 5 for each group. **P < 0.01 or *P < 0.05 vs. the control group. **c-d** Analysis of hepatic VLDL synthesis and MTTP expression. Post-Triton VLDL production rate in fasting rabbits at 8 weeks after HFFD or chow diet feeding was measured. Blood was drawn at 0 minutes (before administration of Triton WR-1339) and 2, 4 and 6 hours after Triton WR-1339 injection. Triglyceride contents of total plasma (C, top) and VLDLs (d <1.006 *g*/mL) (C, bottom) were quantified. Hepatic mRNA expression of microsomal triglyceride transfer protein (MTTP) was analyzed using real-time RT-PCR (D). Data are expressed as the mean ± SD. n = 5 for each group. *P < 0.05 vs. the control group

Moreover, HFFD-fed rabbits had higher plasma TC and TG levels (Fig. 1) than control rabbits throughout the experimental period, but their HDL-C levels were unchanged (data not shown). Analysis of lipoprotein profiles by HPLC and ultracentrifugation revealed that increased plasma TC and TG levels in the HFFD group were attributable to increased apolipoprotein (apo)-B containing particles, very-low-density lipoproteins (VLDLs) and LDLs because both TC and TG contents in these fractions were significantly increased compared with those of control rabbits (Fig. 2a and Additional file 1: Figure S2). Quantitative analysis of LDL particles showed that the levels of all LDL particles were increased in the HFFD group, especially those of small-sized LDL particles (Fig. 2b). To illustrate the possible mechanisms responsible for increased plasma VLDLs and LDLs induced by HFFD, we measured the rate of hepatic VLDL secretion *in vivo* using Triton WR-1339 to block the hydrolysis of TG-rich lipoproteins by lipoprotein lipase [18]. As shown in Fig. 2c-d, HFFD increased the VLDL synthesis rate, which was accompanied by higher expression of MTTP mRNA, the rate-limiting mediator for VLDL production in the liver.

### Effect of HFFD on glucose and insulin metabolism

**Fig. 3:**
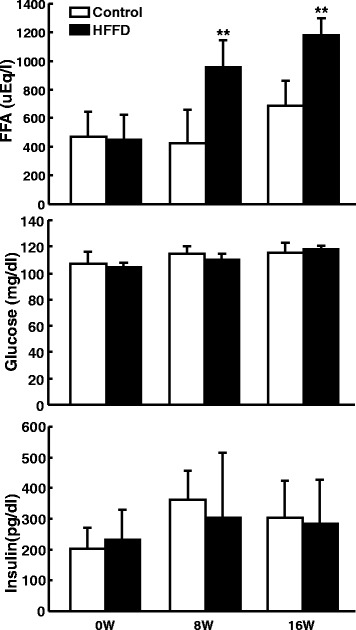
Plasma concentrations of free fatty acids, glucose, and insulin. Plasma was collected from fasted animals and measured as described in the Methods. Data are expressed as the mean ± SD. n = 5-10 for each group. **P < 0.01 vs. the control group

**Fig. 4:**
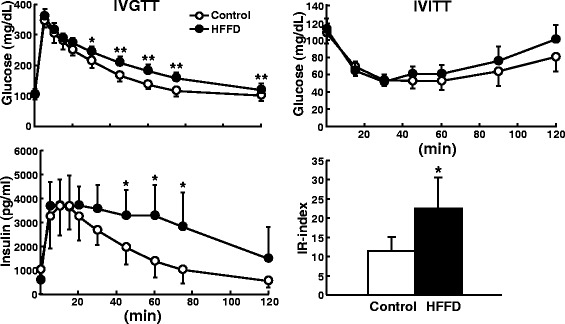
Evaluation of glucose and insulin metabolism by IVGTT and IVITT. Rabbits at 4 weeks after HFFD feeding were intravenously injected with either glucose (left) or insulin (right) as described in the Methods. The changes in plasma glucose levels (top left and right) and insulin levels (bottom left) were determined. Insulin resistance index (IR-index) was calculated [19]. Data are expressed as the mean ± SD. n = 6 for each group. **P < 0.01 or *P < 0.05 vs. the control group

HFFD feeding increased the plasma levels of free fatty acids but did not change the plasma glucose and insulin levels compared with those in the control (Fig. 3). In addition, HFFD led to impaired glucose and insulin response. As shown in Fig. 4, HFFD-fed rabbits exhibited delayed capacity for clearing glucose from the circulation when challenged with a glucose bolus, which was accompanied by higher insulin secretion and reduced insulin response. This reduced response to insulin induced by HFFD was first noted at 4 weeks and continued at 8 and 16 weeks (Additional file 1: Figure S3). However, the glucose intolerance did not get worse over the course (4, 6, 8 weeks), suggesting that aging may influence glucose tolerance in WHHL rabbits.

Then, we examined the histological changes of the pancreas and found that islet number and area of the pancreas of the HFFD group were significantly increased by 58 % and 65 % compared with those of the control at 8 weeks (Additional file 1: Figure S4). Taken together, these results showed that the HFFD group exhibited insulin resistance.

### Pathological examination of liver and adipose tissue

Pathological examinations revealed that the liver of HFFD-fed rabbits showed hepatic steatosis while the liver weight was unchanged (Additional file 1: Figure S5). At 8 weeks, 7 of 9 HFFD-fed rabbits (77 %) showed remarkable fatty liver, in contrast to control rabbits, in which only 2 of 10 rabbits (20 %) had mild and focal hepatic steatosis (Additional file 1: Figure S5), and similar results were obtained at 16 weeks (data not shown). We next examined oxidative stress-associated gene expression in the liver using real-time RT-PCR and found that Nrf2 mRNA expression was increased whereas SOD1 was reduced in HFFD-fed rabbits compared to the control (Additional file 1: Figure S5). In addition, plasma levels of TBARS were significantly increased in HFFD-fed group (Additional file 1: Figure S5).

**Fig. 5:**
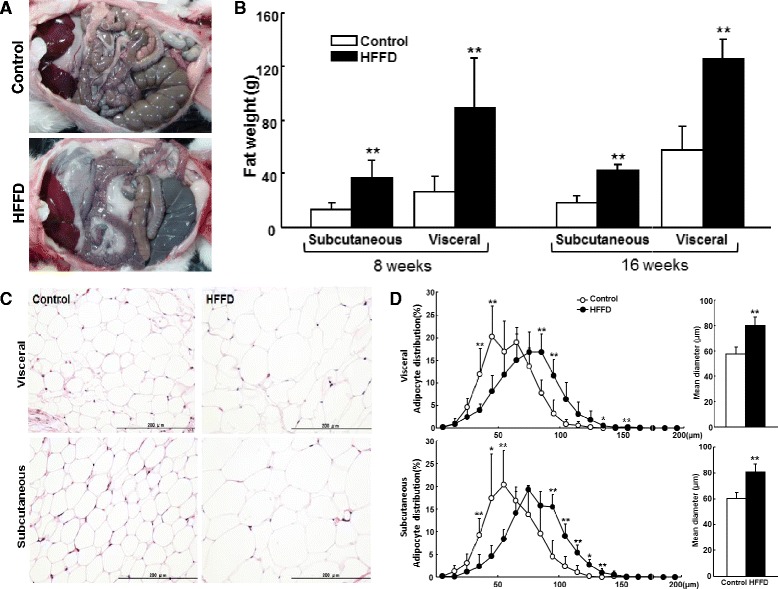
Pathological analysis of adipose tissue. **a** Representative pictures of visceral adipose accumulation of each group at 16 weeks. **b** Total mass of subcutaneous and visceral adipose tissue was collected and measured. Data are expressed as the mean ± SD. n = 9-10 for each group at 8 weeks and 5 for each group at 16 weeks. **P < 0.01 or *P < 0.05 vs. the control group. **c** Representative micrographs of adipose tissue stained with H&E. The HFFD group (right) showed hypertrophic (larger) adipocytes compared with the control group (left). **d** Morphometric analyses of adipocyte cell size distribution and mean diameter were determined using the specimens obtained from rabbits at 16 weeks. Data are expressed as the mean ± SD. n = 5 for each group. **P < 0.01 or *p < 0.05 vs. the control group

We next examined adipose tissue and found that the amount of adipose accumulation of the HFFD group was significantly increased in both subcutaneous (1.8- and 1.28-fold increases over the control at 8 and 16 weeks) and visceral regions (2.37- and 1.21-fold increases over the control at 8 and 16 weeks) (Fig. 5a-b), although their body weights were similar (Additional file 1: Figure S1). Histological and morphometric analysis showed that adipose tissue of the HFFD group was characterized by an increased number of large-sized adipocytes, which leads to the cell size distribution being shifted towards a larger size in both visceral and subcutaneous regions (Fig. 5c-d). We made a thorough evaluation of adipose tissue histology and did not find prominent increase of macrophage infiltration in the HFFD group compared to the control group. This may be not surprising because HFFD feeding did not induce remarkable obesity in rabbits.

We also measured blood pressure and heart rate [20], but did not find any differences between the two groups (Additional file 1: Figure S6).

### Aortic atherosclerosis

**Fig. 6:**
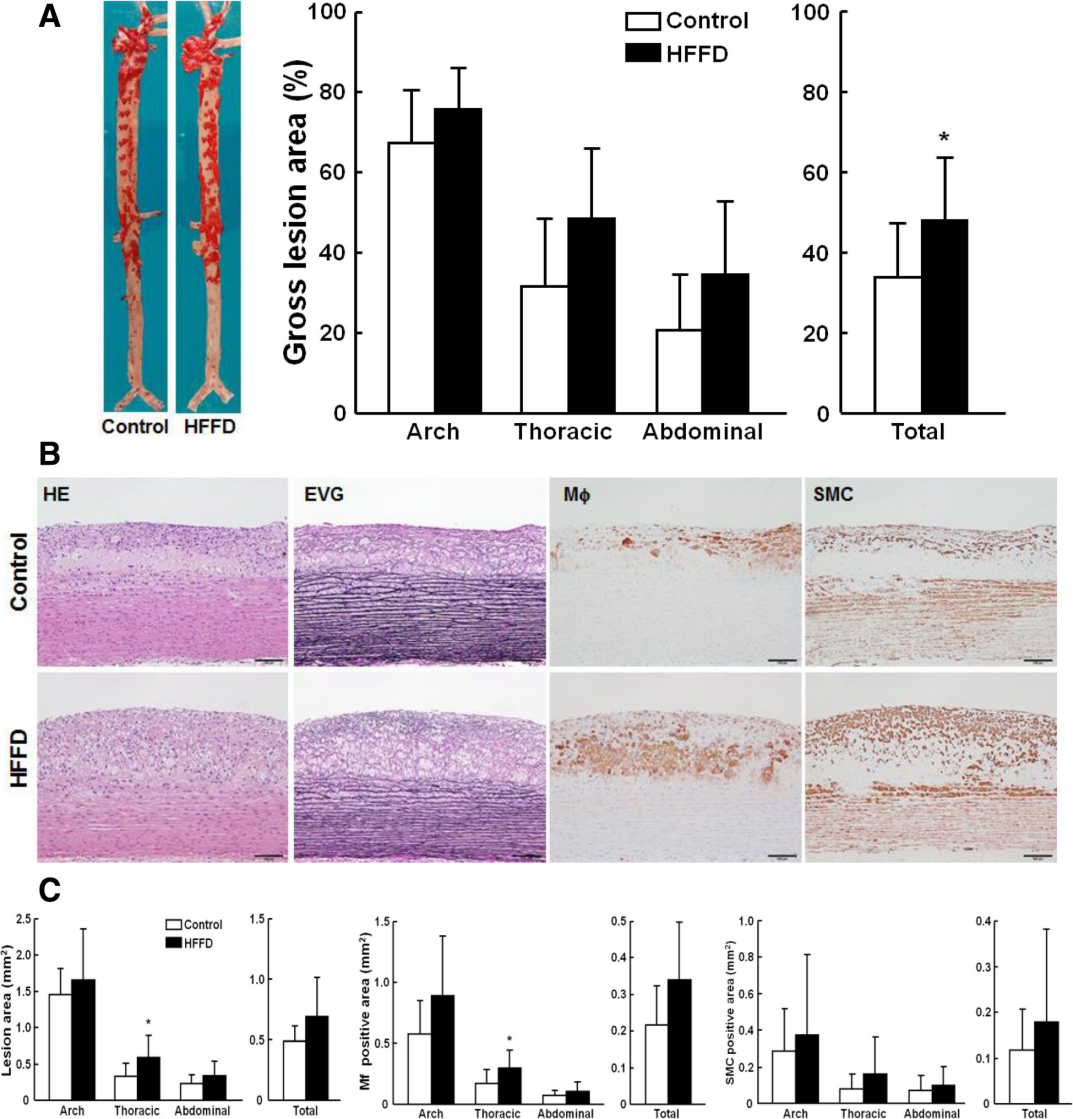
Quantitation of aortic atherosclerosis at 8 weeks after HFFD feeding. **a** Representative pictures of aortas stained with Sudan IV are shown on the left. The lesion area (defined by sudanophilic staining as red) was quantified using an image analysis system (right). Data are expressed as the mean ± SD. n = 9-10 for each group. *P < 0.05 vs. the control group. **b** Representative micrographs of the aortic arch lesions from each group. Serial paraffin sections were stained with hematoxylin-eosin (HE) and elastica van Gieson (EVG) or immunohistochemically stained with mAbs against either macrophages (Mϕ) or α-smooth muscle actin for smooth muscle cells (SMCs). The lesions are characterized by intimal accumulation of macrophage-derived foam cells intermingled with smooth muscle cells. **c** Quantitation of the lesions of different parts of aortas. The intimal lesion area and positively immunostained area of macrophages and SMCs were quantified using an image analysis system as described in the Methods. Data are expressed as the mean ± SD. n = 9-10 for each group. *P < 0.05 vs. the control group

At 8 weeks, *en face* lesion area was increased by 40 % (p < 0.0.5) in the whole aorta of the HFFD group compared with that in the controls (Fig. 6a), with 11 % (p = 0.14), 60 % (p = 0.052), and 70 % (p = 0.08) increase in aortic arch, thoracic and abdominal aorta, but the differences were not statistically significant. Histological examination along with immunohistochemical staining showed that the atherosclerotic lesions of both HFFD and control groups were mainly composed of fatty streaks (Fig. 6b); however, the microscopic lesion size of the total aorta was increased in the HFFD group due to increased numbers of both macrophages (55 % increase over the control) and smooth muscle cells (54 % increase over the control), even though this was not statistically significant (p = 0.05) (Fig. 6c).

**Fig. 7:**
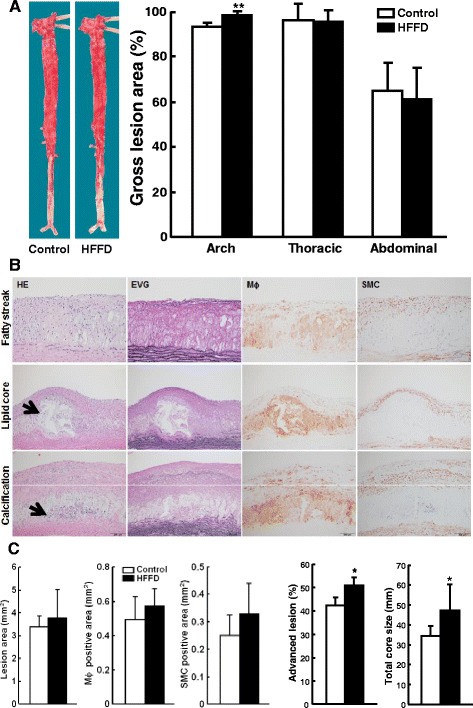
Quantitation of aortic atherosclerosis at 16 weeks after HFFD feeding. **a** Representative pictures of aortas stained by Sudan IV are shown on the left. The lesion area (defined by sudanophilic staining as red) was quantified using an image analysis system (right). Data are expressed as the mean ± SD. n = 5 for each group. **P < 0.01 vs. the control group. **b** Representative micrographs of the aortic arch lesions from each group. Serial paraffin sections were stained with hematoxylin-eosin (HE) and elastica van Gieson (EVG) or immunohistochemically stained with mAbs against either macrophages (Mϕ) or α-smooth muscle actin for smooth muscle cells (SMCs). Histologically, there are two types of lesion: (1) early-stage lesions (fatty streaks) are mainly composed of foam cells as shown at 8 weeks above (top); and (2) advanced lesions are characterized by the formation of lipid core or necrotic cores covered by a fibrous cap. In addition, calcification is often seen (middle and bottom indicated by arrowheads). **c** Quantitation of aortic arch lesions, macrophages and smooth muscle cells, the early stage lesions, and advanced lesions. Data are expressed as the mean ± SD. n = 5 for each group. *P < 0.05 vs. the control group

At 16 weeks, the aortic surfaces of both groups were almost completely covered by the atherosclerotic lesions, while only the lesion area of the aortic arch of HFFD group was greater than that of the control group (p < 0.01) (Fig. 7a). In contrast to the lesions at 8 weeks, in which early-stage lesions are predominant as described above, the lesions at 16 weeks of both groups became more advanced and many parts of the aortas showed necrotic or lipid core formation and calcification in addition to fatty streaks (Fig. 7b). Similarly to lesions at 8 weeks, HFFD feeding for 16 weeks increased microscopic lesion, with tendencies for increasing macrophages, and smooth muscle cells although these differences were not statistically significant (Fig. 7c). Nevertheless, the advanced lesions and the lipid core size of HFFD-fed WHHL rabbits were significantly increased compared to the control group (Fig. 7c).

### Coronary atherosclerosis

**Fig. 8:**
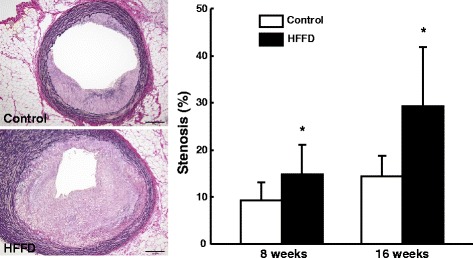
Quantitation of coronary atherosclerosis. Representative micrographs of the left coronary lesions from each group (EVG staining) are shown on the left and the lesion size (expressed as stenosis %) of both right and left coronary arteries is shown on the right. Data are expressed as the mean ± SD. n = 9-10 for each group at 8 weeks and 5 for each group at 16 weeks. *P < 0.05 vs. the control group

In addition to the aorta, the HFFD led to a significant increase of coronary atherosclerosis. There was a 60 % increase of coronary stenosis (both left and right coronary arteries) at 8 weeks and a 103 % increase at 16 weeks compared with that of the controls (Fig. 8).

## Discussion

In the current study, we fed WHHL rabbits with two kinds of diets: HFFD (rich in sugar and fat with reduced protein and fibers) and standard chow diet (protein- and fiber-rich). Although both groups consumed an equal amount of calorie of each diet, HFFD feeding led to prominent IR accompanied by elevated plasma lipids, hepatic steatosis and adipose accumulation, even though the body weight was unchanged. Increased plasma levels of lipids are basically caused by high uptake of free fatty acids into the liver where they can be synthesized into VLDLs, which are accumulated in the plasma. At the same time, VLDL accumulation was further enhanced due to delayed catabolism of VLDLs in the context of deficiency of LDL receptors in WHHL rabbits. It should be pointed out that high-fat diet feeding did not increase plasma lipids in wild-type rabbits which have normal LDL receptors [20]. This notion is further supported by our Triton experiments along with RT-PCR analysis showing that there was increased production of VLDLs and expression of hepatic MTTP mRNA in HFFD-fed WHHL rabbits. Increased free fatty acids in the circulation can also induce fatty liver and adipose accumulation, and reduce the response to insulin thereby causing IR [3]. A high flux of fructose to the liver, the main organ capable of metabolizing this simple carbohydrate, perturbs glucose metabolism and glucose uptake pathways, and leads to a significantly enhanced rate of *de novo* lipogenesis and TG synthesis, driven by the high flux of glycerol and acyl portions of TG molecules from fructose catabolism. This conjecture is implicated by the finding that HFFD-fed WHHL rabbits had prominent fatty liver compared with the control group. In addition, there was increased Nrf2 expression accompanied by low SOD1 expression in the liver, suggesting that HFFD may induce hepatic insulin resistance and subsequent steatosis through the enhancement of increased free fatty acid influx into the portal system and hepatic oxidative stress [21, 22].

Secondly, HFFD-fed WHHL rabbits had greater aortic and coronary atherosclerosis than the control WHHL rabbits. In the current study, we compared the lesions of atherosclerosis after feeding on HFFD for 8 and 16 weeks in an attempt to investigate the short-term effects of HFFD-induced IR on the early-stage lesions as well as the long-term effects of HFFD-induced IR on the advanced lesions of atherosclerosis. At 8 weeks, aortic atherosclerosis was increased by 40 % in the HFFD group compared with that in the control group and the lesions were characterized by increased macrophages and smooth muscle cells, suggesting that HFFD feeding affects the cellular components in the early-stage lesions. Interestingly, at 16 weeks, aortic surface atherosclerotic lesions became saturated due to the presence of hypercholesterolemia in both groups, so the differences of the total aortic lesion size between HFFD groups and controls are not as prominent as those shown at 8 weeks. In spite of this, the lesions at 16 weeks are characterized by increased advanced lesions, such as lipid cores and calcification. Increased lipid cores and calcification in the lesions of the HFFD group indicate that the lesions become more vulnerable and more susceptible to complications. In the current study, we also demonstrated that HFFD induced the enhancement of coronary stenosis. It should be pointed out that; however, enhanced aortic and coronary atherosclerosis induced by HFFD occurs through several mechanisms, among which IR may play the major roles. For example, IR can be an antecedent or cause of elevated levels of plasma lipids, adipose tissue accumulation and fatty liver as described above. It is also likely that these disorders exhibit a deleterious effect on insulin sensitivity thereby enhancing atherosclerosis. Therefore, it is conceivable that IR together with elevated plasma lipids and adipose accumulation [20, 23, 24] exhibits combinational roles in enhancing the progression of atherosclerosis in HFFD-fed WHHL rabbits. In addition, there is an increase of plasma LDLs, especially small-sized LDLs, in HFFD-fed WHHL rabbits, which is often seen in metabolic syndrome patients. It has been shown that small LDLs are more atherogenic than large ones because they are trapped in the arterial intima more easily and become oxidized [25, 26]. Unlike WHHL rabbits, fructose-fed LDL receptor-deficient mice failed to develop IR while their aortic atherosclerosis was greater than Western-type diet-fed counterpart mice in which IR was present [8]. Apparently, LDL receptor-deficient mice are different from WHHL rabbits in terms of lipid metabolism and susceptibility to atherosclerosis as shown in the current study.

## Conclusion

We have shown that HFFD-fed WHHL rabbits showed enhancement of the atherosclerosis. The lesions of HFFD-fed WHHL rabbits are rich in macrophages at the early stage and characterized by increased lipid core formation in the advanced lesions. This finding is of clinical importance because our results suggest that even without apparent obesity as shown in the current study, HFFD (even on a normal calorie range) can be still harmful to insulin sensitivity thereby affecting atherosclerosis.

## Additional file

Additional file 1:
**Table S1.** Primers used for Real-Time RT-PCR. **Table S2.** Average daily food and calorie intake. **Figure S1.** Food intake, calorie intake and body weight of WHHL rabbits are shown. **Figure S2.** Analysis of lipoprotien density fractions. **Figure S3.** IVGTT and IVITT measured at 8 weeks and 16 weeks. **Figure S4.** Immunohistochemical staining and morphometric analyses of the islet size and area of pacreas. **Figure S5.** Micrographs of the liver (upper left), liver weight (upper right), hepatic mRNA expression of Nfr2 and SOD1 (lower left and middle) and plasma level of TBARS (bottom right) are shown. **Figure S6.** Blood pressure and heart rate (beat/min) were measured at 16 weeks using the method described in Ref. 13. (PDF 545 kb)

## Abbreviations

CETP: Cholesteryl ester transfer protein
EVG: Elastica van Gieson
HDL-C: High density lipoprotein-cholesterol
H&E: Hematoxylin and eosin
HFFD: High fat and fructose diet
HPLC: High-performance liquid chromatography
IR: Insulin resistance
IVGTT: Intravenous glucose tolerance test
IVITT: Intravenous insulin tolerance test
LDL: Low density lipoprotein
MTTP: Microsomal triglyceride transfer protein
Nrf2: Nuclear factor-like 2
RT-PCR: Reverse transcription polymerase chain reaction
SMC: Smooth muscle cell
SOD1: Superoxide dismutase 1
TC: Total cholesterol
TG: Triglycerides
VLDL: Very low density lipoprotein
WHHL rabbits: Watanabe heritable hyperlipidemic rabbits
