# Supplementary material for: High-fructose and high-fat diet-induced insulin resistance enhances atherosclerosis in Watanabe heritable hyperlipidemic rabbits
Source: Nutr Metab (Lond). 2015 Aug 12;12:30. doi: 10.1186/s12986-015-0024-3 (PMC4531510; doi:10.1186/s12986-015-0024-3)
Supplement: Additional file 1: — Table S1. Primers used for Real-Time RT-PCR. Table S2. Average daily food and calorie intake. Figure S1. Food intake, calorie intake and body weight of WHHL rabbits are shown. Figure S2. Analysis of lipoprotien density fractions. Figure S3. IVGTT and IVITT measured at 8 weeks and 16 weeks. Figure S4. Immunohistochemical staining and morphometric analyses of the islet size and area of pacreas. Figure S5. Micrographs of the liver (upper left), liver weight (upper right), hepatic mRNA expression of Nfr2 and SOD1 (lower left and middle) and plasma level of TBARS (bottom right) are shown. Figure S6. Blood pressure and heart rate (beat/min) were measured at 16 weeks using the method described in Ref. 13. (PDF 545 kb) [file 12986_2015_24_MOESM1_ESM.pdf]

S-Table 1: Primers used for Real-Time RT-PCR

Gene		Primers
MTTP	Forward	GTCTTCCAGAGCGAGTGTAAAG
	Reverse	GGCAAATCCACAGGCATAAAG
Nrf2	Forward	CCCAACACATCCAGTCAGAAA
	Reverse	TCAGCTATGAAGGCAGCATAAA
SOD1	Forward	GGTGTCGCTGATGTGTTAATTG
	Reverse	GCAGGTGACAGTGTTTAATGTG
GAPDH	Forward	ATCACTGCCACCCAGAAGAC
	Reverse	GTGAGTTTCCCGTTCAGCTC

S-Table 2: Average daily food and calorie intake

	Control (n=15)	HFFD (n=14)
**Food intake (g/day)**	**73 ± 9**	**62 ± 8**
**Calorie intake (kcal/day)**	**219 ± 29**	**221 ± 27**

Data are expressed as the mean ± SD

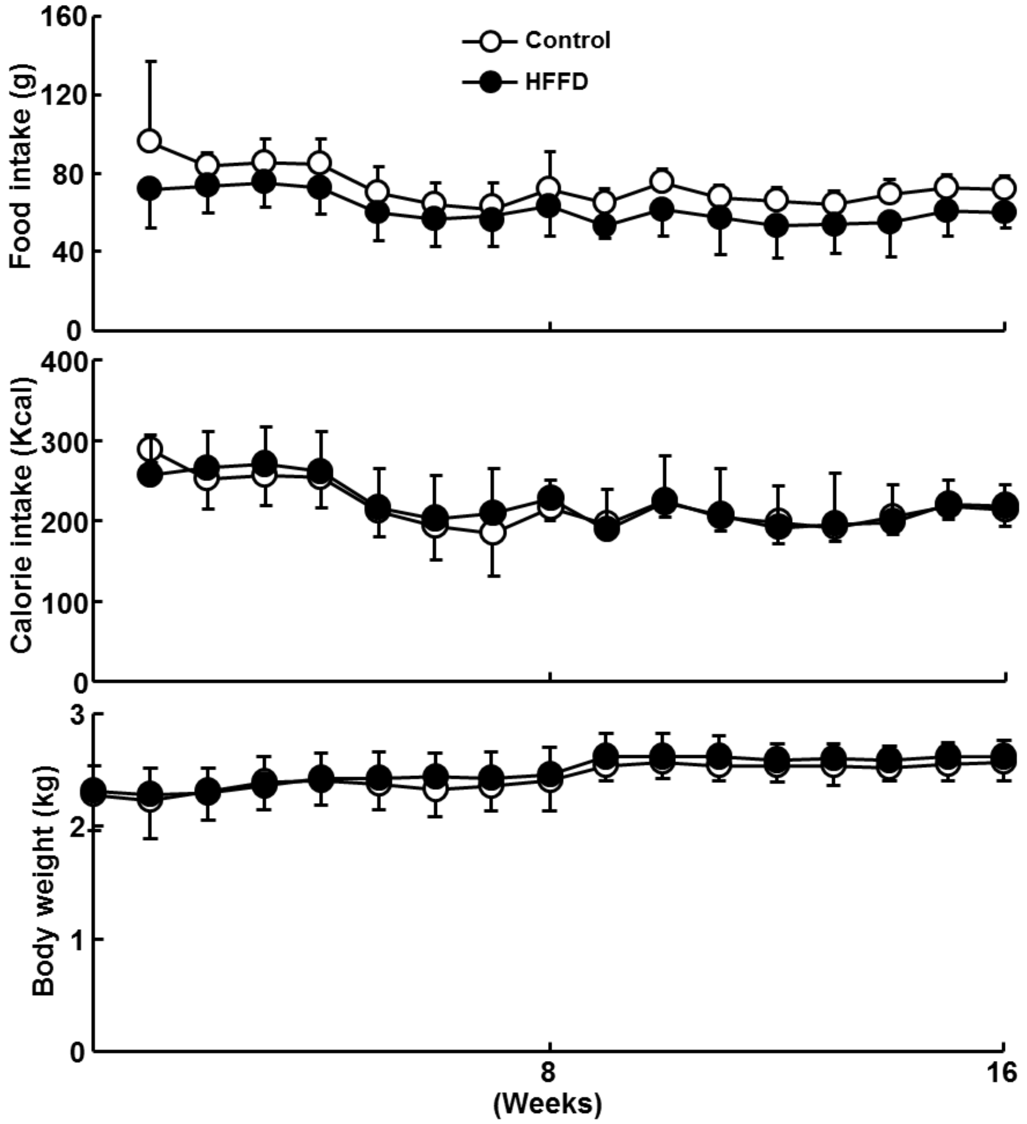

S-Figure 1. Food intake, calorie intake and body weight of WHHL rabbits are shown. Data are expressed as the mean ± SD. n=5-15 for each group.

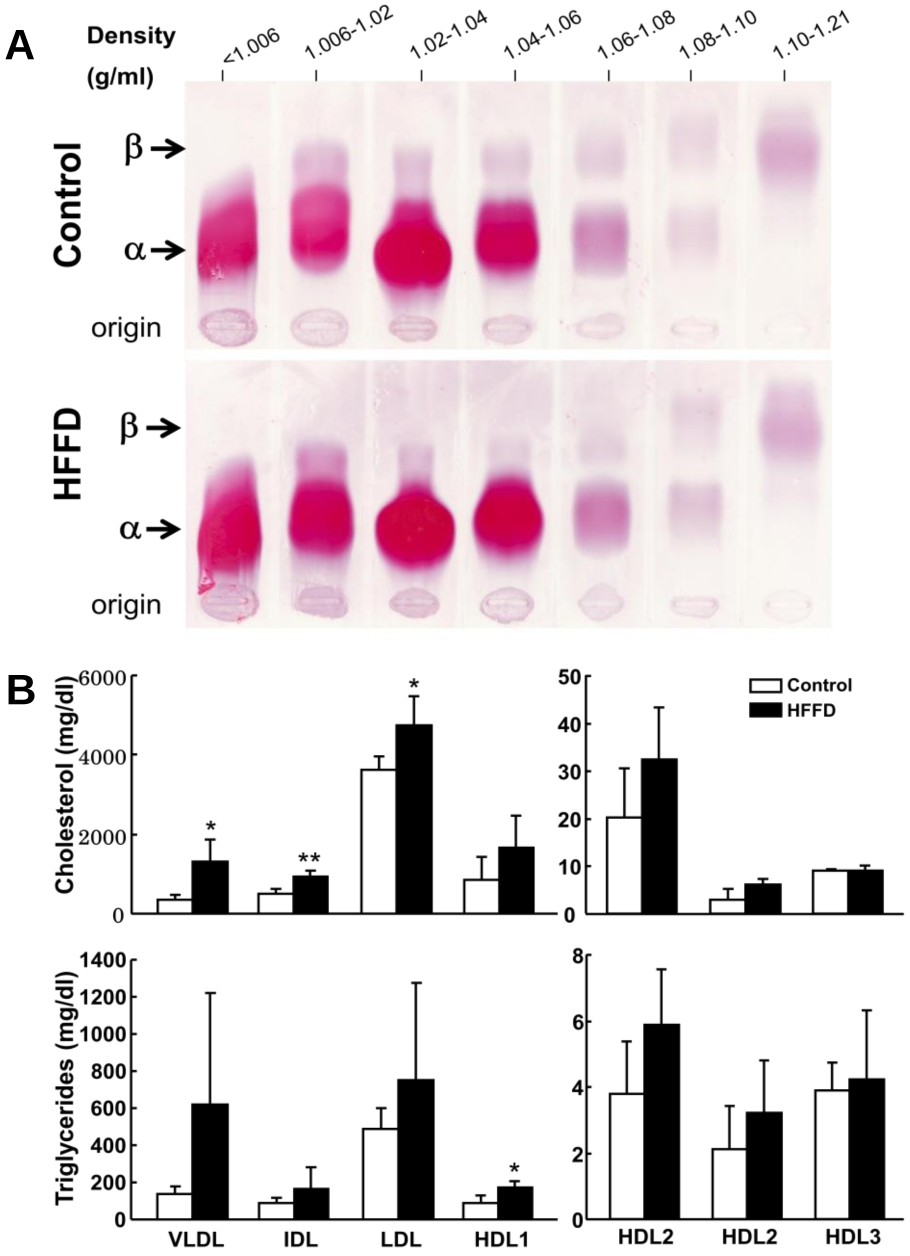

S-Figure 2. Analysis of lipoprotein density fractions. Plasma lipoproteins from HFFD and control groups were separated by sequential density ultracentrifugation using the density ranges shown as agarose gels. Equal volumes of each fraction (8 μl) were resolved by electrophoresis in a 1% agarose gel. Lipoproteins were visualized using Fat Red 7B staining (A). The electrophoretic mobility of lipoprotein classes is indicated by arrows. The quantification of TC and TG contents in each fraction is shown (B). Data are expressed as the mean ± SD. n=4 for each group. ***P*<0.01 or **p* < 0.05 vs. the control group.

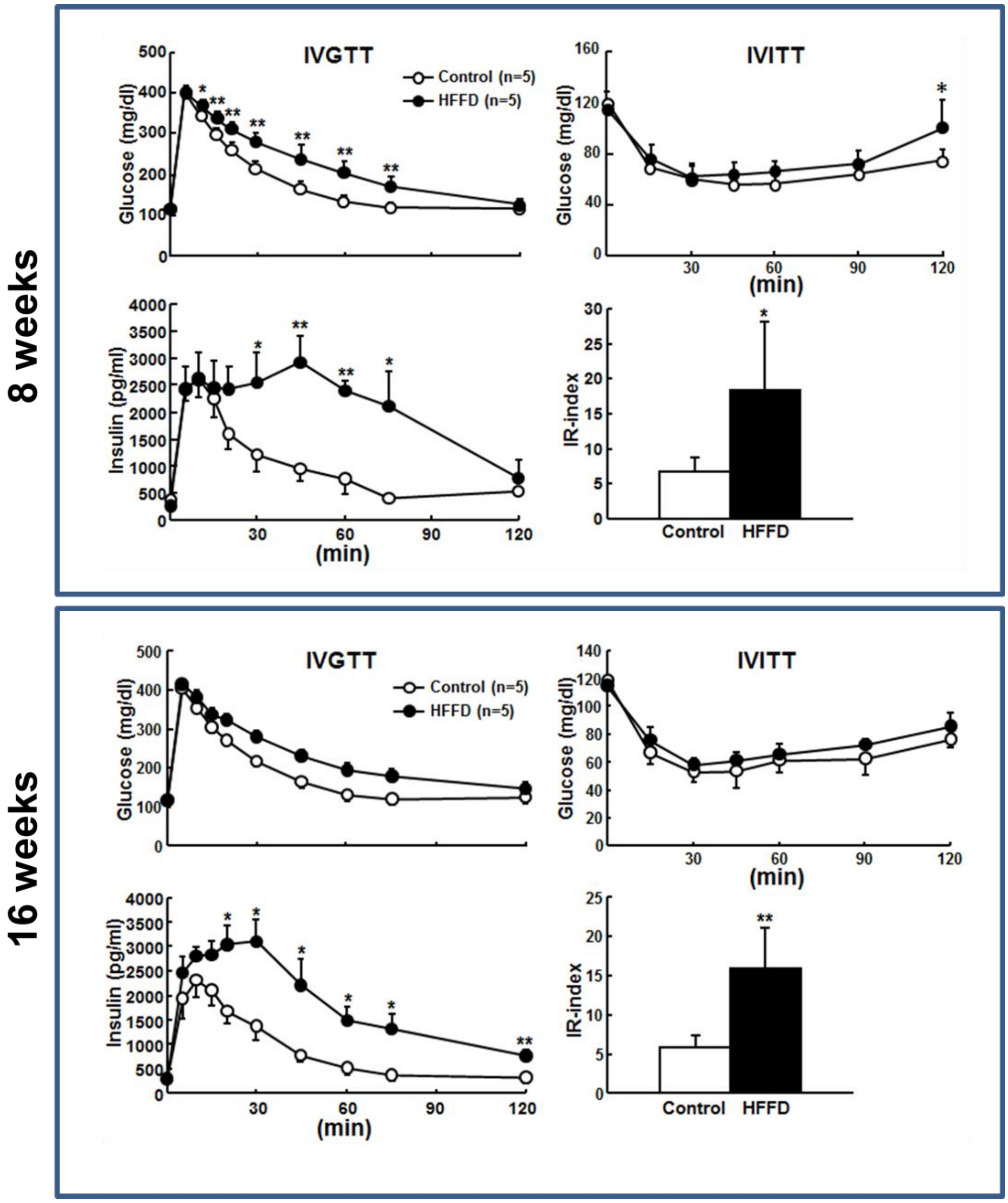

S-Figure 3. IVGTT and IVITT measured at 8 weeks and 16 weeks. Data are expressed as the mean ± SD. n=5 for each group. ***P*<0.01 or **p* < 0.05 vs. the control group.

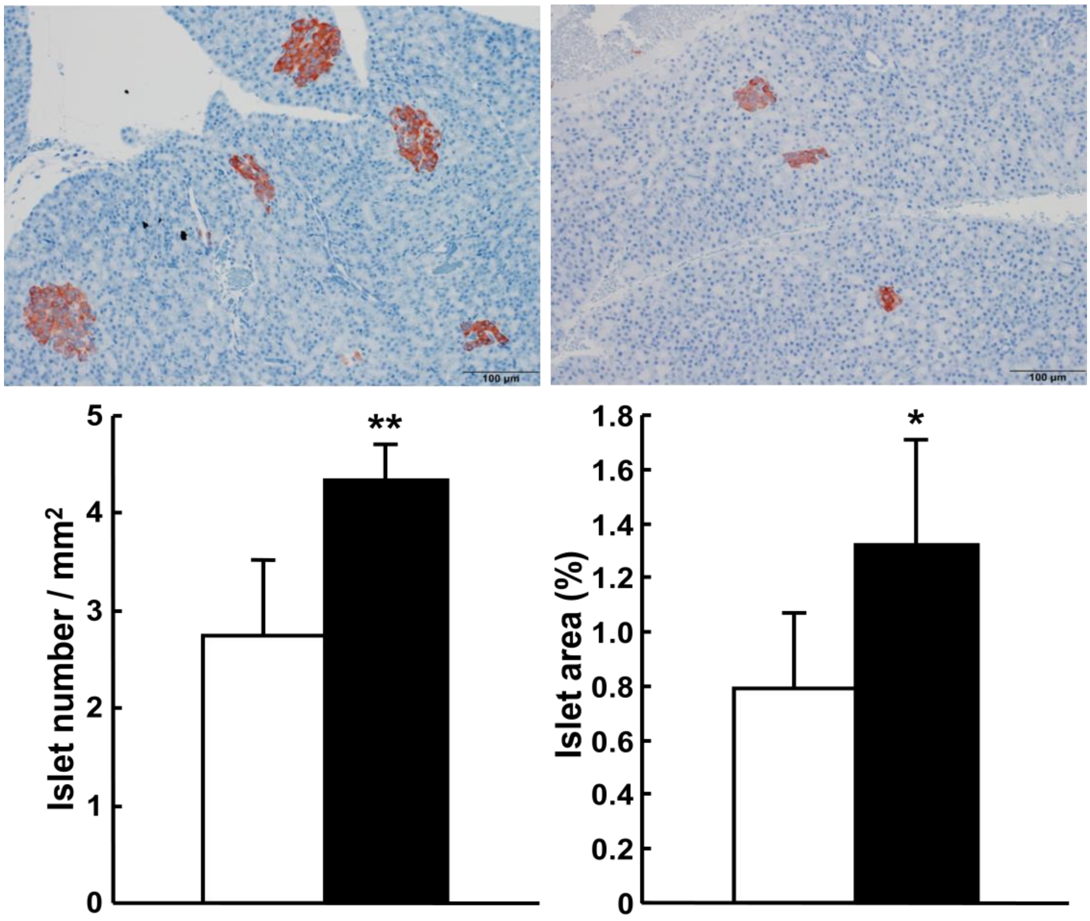

S-Figure 4. Immunohistochemical staining and morphometric analyses of the islet size and area of the pancreas. Paraffin-sections were immunohistochemically stained with monoclonal antibody against insulin (upper panel). The islet number and area are measured using image analysis system (bottom panel). Data are expressed as the mean ± SD. n=9-10 for each group. **P<0.01 or *p < 0.05 vs. the control group.

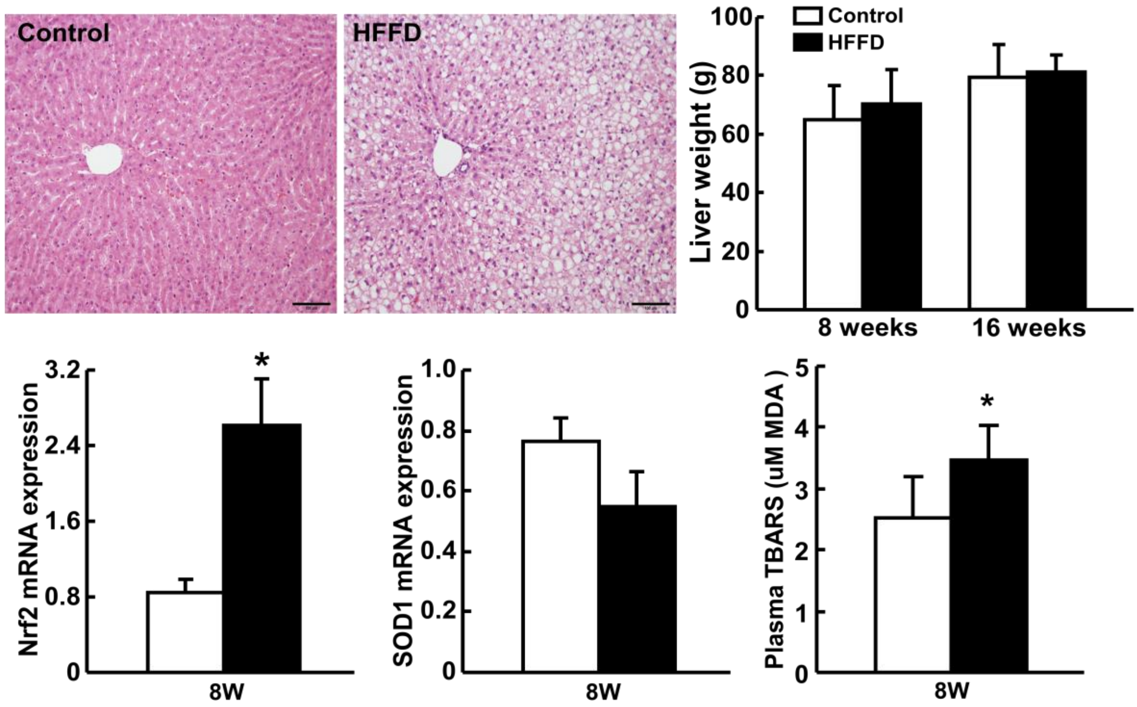

S-Figure 5. Micrographs of the liver (upper left), liver weight (upper right), hepatic mRNA expression of Nrf2 and SOD1 (lower left and middle) and plasma level of TBARS (bottom right) are shown. Data are expressed as the mean ± SD. n=4-10 for each group. *p < 0.05 vs. the control group. MDA: malondialdehyde.

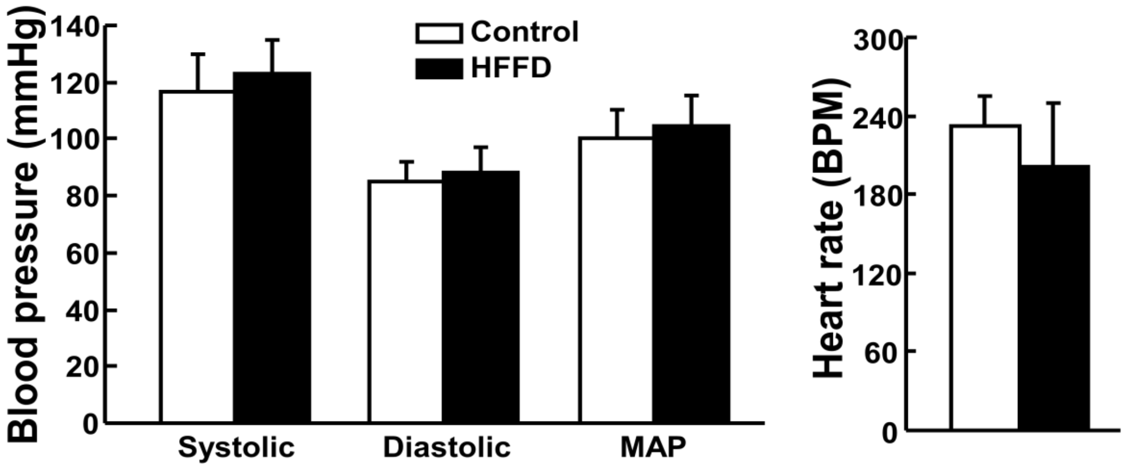

S-Figure 6. Blood pressure and heart rate (beat/min) were measured at 16 weeks using the method described in Ref.13. Data are expressed as the mean ± SD. N=5 for each group.
